# Impact of species and antibiotic therapy of enterococcal peritonitis on 30-day mortality in critical care—an analysis of the OUTCOMEREA database

**DOI:** 10.1186/s13054-019-2581-8

**Published:** 2019-09-06

**Authors:** Anne-Cécile Morvan, Baptiste Hengy, Maïté Garrouste-Orgeas, Stéphane Ruckly, Jean-Marie Forel, Laurent Argaud, Thomas Rimmelé, Jean-Pierre Bedos, Elie Azoulay, Claire Dupuis, Bruno Mourvillier, Carole Schwebel, Jean-François Timsit, On behalf of the OUTCOMEREA study group, Jean-François Timsit, Elie Azoulay, Maïté Garrouste-Orgeas, Jean-Ralph Zahar, Bruno Mourvillier, Michael Darmon, Christophe Clec’h, Corinne Alberti, Stephane Ruckly, Sébastien Bailly, Aurélien Vannieuwenhuyze, Romain Hernu, Christophe Adrie, Carole Agasse, Bernard Allaouchiche, Olivier Andremont, Pascal Andreu, Laurent Argaud, Claire Ara-Somohano, Elie Azoulay, Francois Barbier, Déborah Boyer, Jean-Pierre Bedos, Thomas Baudry, Jérome Bedel, Julien Bohé, Lila Bouadma, Jeremy Bourenne, Noel Brule, Cédric Brétonnière, Frank Chemouni, Christine Cheval, Julien Carvelli, Elisabeth Coupez, Martin Cour, Claire Dupuis, Etienne de Montmollin, Loa Dopeux, Anne-Sylvie Dumenil, Jean-Marc Forel, Marc Gainnier, Charlotte Garret, Dany Goldgran-Tonedano, Steven Grangé, Antoine Gros, Hédia Hammed, Akim Haouache, Romain Hernu, Tarik Hissem, Vivien Hong Tuan Ha, Sébastien Jochmans, Jean-Baptiste Joffredo, Hatem Kallel, Guillaume Lacave, Virgine Laurent, Alexandre Lautrette, Clément Le bihan Eric Magalhaes, Virgine Lemiale, Guillaume Marcotte, Jordane Lebut, Maxime Lugosi, Sibylle Merceron, Benoît Misset, Mathild Neuville, Laurent Nicolet, Johanna Oziel, Laurent Papazian, Juliette Patrier, Benjamin Planquette, Aguila Radjou, Marie Simon, Romain Sonneville, Jean Reignier, Bertrand Souweine, Carole Schwebel, Shidasp Siami, Romain Sonneville, Nicolas Terzi, Gilles Troché, Marie Thuong, Guillaume Thierry, Marion Venot, Sondes Yaacoubi, Olivier Zambon, Julien Fournier, Stéphanie Bagur, Mireille Adda, Vanessa Vindrieux, Sylvie de la Salle, Pauline Enguerrand, Vincent Gobert, Stéphane Guessens, Helene Merle, Nadira Kaddour, Boris Berthe, Samir Bekkhouche, Kaouttar Mellouk, Mélaine Lebrazic, Carole Ouisse, Diane Maugars, Christelle Aparicio, Igor Theodose, Manal Nouacer, Veronique Deiler, Fariza Lamara, Myriam Moussa, Atika Mouaci, Nassima Viguier

**Affiliations:** 1 Department of Anesthesiology and Critical Care Medicine, Hospices Civils de Lyon, Edouard Herriot Teaching Hospital, 5 place d’Arsonval, 69003 Lyon, France; 2 Polyvalent ICU, St Joseph Hospital, Paris, France; 3 UMR 1137 – IAME Team 5 – DeSCID: Decision Sciences in Infectious Diseases, Control and Care INSERM Paris Diderot University, Sorbonne Paris Cité, Paris, France; 4 Medical ICU, Respiratory Distress and Severe Infections, Nord Hospital, URMITE CNRS-UMR 6236, Aix-Marseille University, AP-HM, Marseille, France; 5 Medical ICU, Hospices Civils de Lyon, Edouard Herriot Teaching Hospital, Lyon, France; 6 Intensive Care Department, GHT Sud Yvelines, Centre Hospitalier de Versailles - Site André Mignot, Le Chesnay, Cedex France; 7 Medical ICU, APHP, Saint-Louis Hospital, ECSTRA Team, and Clinical Epidemiology, UMR 1153 (Center of Epidemiology and Biostatistics, Sorbonne Paris Cité, CRESS), INSERM, Paris Diderot Sorbonne University, Paris, France; 8 Medical and Infectious Diseases ICU, Bichat University Hospital, AP-HP, Paris, France; 9 Medical ICU, Albert Michallon Hospital, Grenoble 1 University, Grenoble, France

**Keywords:** Intensive care, Intraabdominal infections, Mortality, *Enterococcus* spp*.*, Antibiotic therapy

## Abstract

**Introduction:**

*Enterococcus* species are associated with an increased morbidity in intraabdominal infections (IAI). However, their impact on mortality remains uncertain. Moreover, the influence on outcome of the appropriate or inappropriate status of initial antimicrobial therapy (IAT) is subjected to debate, except in septic shock. The aim of our study was to evaluate whether an IAT that did not cover *Enterococcus* spp. was associated with 30-day mortality in ICU patients presenting with IAI growing with *Enterococcus* spp.

**Material and methods:**

Retrospective analysis of French database OutcomeRea from 1997 to 2016. We included all patients with IAI with a peritoneal sample growing with *Enterococcus*. Primary endpoint was 30-day mortality.

**Results:**

Of the 1017 patients with IAI, 76 (8%) patients were included. Thirty-day mortality in patients with inadequate IAT against *Enterococcus* was higher (7/18 (39%) vs 10/58 (17%), *p* = 0.05); however, the incidence of postoperative complications was similar. Presence of *Enterococcus* spp. other than *E. faecalis* alone was associated with a significantly higher mortality, even greater when IAT was inadequate. Main risk factors for having an *Enterococcus* other than *E. faecalis* alone were as follows: SAPS score on day 0, ICU-acquired IAI, and antimicrobial therapy within 3 months prior to IAI especially with third-generation cephalosporins. Univariate analysis found a higher hazard ratio of death with an *Enterococcus* other than *E. faecalis* alone that had an inadequate IAT (HR = 4.4 [1.3–15.3], *p* = 0.019) versus an adequate IAT (HR = 3.1 [1.0–10.0], *p* = 0.053). However, after adjusting for confounders (i.e., SAPS II and septic shock at IAI diagnosis, ICU-acquired peritonitis, and adequacy of IAT for other germs), the impact of the adequacy of IAT was no longer significant in multivariate analysis. Septic shock at diagnosis and ICU-acquired IAI were prognostic factors.

**Conclusion:**

An IAT which does not cover *Enterococcus* is associated with an increased 30-day mortality in ICU patients presenting with an IAI growing with *Enterococcus*, especially when it is not an *E. faecalis* alone. It seems reasonable to use an IAT active against *Enterococcus* in severe postoperative ICU-acquired IAI, especially when a third-generation cephalosporin has been used within 3 months.

**Electronic supplementary material:**

The online version of this article (10.1186/s13054-019-2581-8) contains supplementary material, which is available to authorized users.

## Introduction

Intra-abdominal infections (IAI) represent the second most common cause of infection in the ICU [1]. Indeed, they are complicated with septic shock in 40% of cases [2]. Despite improvements in sepsis management, mortality remains high up to 40% in nosocomial IAI [3, 4]. The primary treatment of IAI combines early source control and adequate antimicrobial therapy.

The incidence of Enterococci in IAI is 5 to 20% in community-acquired IAI and 30 to 40% in nosocomial IAI [5]; however, the pathogenicity of Enterococci in IAI is debatable. According to Dupont, Koch, and Fisher, *Enterococcus* might express virulence factors and might synergize with other bacteria like *Escherichia coli* and anaerobes [6–8]. It has been clearly demonstrated that Enteroccoci are associated with proinflammatory responses, greater clinical disease burden, and shock [9–11]. So far, all authors agree with an increase in morbidity (septic shock, higher APACHE 2, and Sequential Organ Failure Assessment (SOFA) scores, higher postoperative infection scores, longer duration of mechanical ventilation and vasopressors, more relaparotomies), but the impact of *Enterococcus* on mortality is unclear [12–16]. Some studies found that the presence of *Enterococci* on peritoneal samples is a predictive factor for death [17–19] whereas others did not [20, 21].

Currently, the impact on prognosis of early antimicrobial therapy against *Enterococcus* spp. is not known and the indication of an initial empiric anti-enterococcal therapy differs among recommendations. Thus, the aim of our study was to compare the role of appropriate versus inappropriate antimicrobial therapy on 30-day mortality in ICU patients with IAI positive for *Enterococcus.*

## Material and methods

This study was a retrospective data analysis from the OUTCOMEREA database (OutcomeRea®).

Data were prospectively collected daily by senior physicians with research assistants in the participating ICUs. All codes and definitions were established prior to study initiation and have been previously described [22]. We collected delay between hospitalization, diagnosis, and surgery.

Patients’ age, sex, and McCabe score were recorded. Severity of illness was evaluated on the first ICU day using the Simplified Acute Physiology Score (SAPS II), Sequential Organ Failure Assessment (SOFA) score, and Glasgow Coma Scale (GCS) score. Knaus’ scale definitions were used to record preexisting chronic organ failures including respiratory, cardiac, hepatic, renal, and immune system failures [23, 24]. Admission category (medical, scheduled surgery, or unscheduled surgery), admission diagnosis (cardiac, respiratory, or neurological failure, infection, and other), invasive procedures (arterial or venous central catheter, Swan-Ganz catheter, or endotracheal intubation), and treatment of organ failures (inotropic support, hemodialysis, and mechanical ventilation) and ICU-acquired infections, bacteriological samples, and daily antimicrobial therapy were also collected.

Data collected from hospitalization records were as follows: risk factors for healthcare-associated infections, antimicrobial therapy within the 3 months prior to ICU admission (particularly with cephalosporins), date of diagnosis of IAI according to clinical, biological and radiological findings, anatomical origin of IAI, localized or generalized type of IAI, community-acquired or nosocomial infection, and pathophysiological mechanisms. We also collected delay between diagnosis according to clinical, biological, and radiological findings and surgery, initial and adapted antimicrobial therapy, *Enterococcus* species, sensibility to antimicrobial therapy, appropriate or inappropriate type of antimicrobial therapy against *Enterococcus* species, day of appropriate antimicrobial therapy for *Enterococcus* species and other microorganisms, surgical complications, need for redo laparotomy or percutaneous drainage, and development of tertiary peritonitis.

### Ethical issue

The database is in accordance with French legislation concerning biomedical research. Patients or their family gave authorization for collection, conservation, and use of their personal anonymized data. Authorizations were obtained from the CNIL (Commission Nationale de l’Informatique et des Libertés), the CCTIRS (Comité consultatif sur le traitement de l’information en matière de recherche), and the Rhône-Alpes-Auvergne Institutional Review Board.

### Inclusion criteria

Adult patients over 18 years old presenting with community-acquired or nosocomial IAI with a peritoneal sample growing with *Enterococcus* spp. and who were admitted to the ICU between 1997 and 2016 were included.

### Non-inclusion criteria

Patients presenting superinfection of necrotizing pancreatitis, missing data either on the *Enterococcus* species or on the antimicrobial therapy, and samples coming from drains in the postoperative period were excluded.

### Definition

*Enterococcus* IAI was defined as an IAI which required surgery or percutaneous drainage and whose intraoperative peritoneal sample was growing with *Enterococcus*. Day 0 (first day of the IAI) was defined as the day of surgery.

Intra-abdominal infections were nosocomial if they appeared after more than 48 h of hospitalization or if there were any risk factor of healthcare-associated infection (hospitalization within 3 previous months, rehabilitation/long-care stay within the 30 previous days, chronic dialysis or chemotherapy within 30 days, or home-care within 30 days).

*Enterococcus* IAI were identified from the infectious data recorded in the database. Medical records were then accessed to confirm diagnosis and details of the surgical procedure.

Initial antimicrobial therapy (IAT) was the antimicrobial therapy started on day 0 or day 1 after surgery. Antimicrobial therapy was considered appropriate or inappropriate according to the antibiogram when available. Otherwise, it was considered appropriate if it used either a penicillin (A or ureido or carboxy) or a carbapenem for *Enterococcus faecalis*, *E. avium*, or *E. durans*, and vancomycin, linezolide, or tigecycline for *Enterococcus faecium*.

Delay between IAI (day 0) and appropriate antimicrobial therapy were extracted from the database separately for *Enterococcus* spp. and other germs including yeasts.

Septic shock was defined according to the Surviving Sepsis Campaign 4th edition (2016) [25]. For septic shock at diagnosis, we considered septic shock criteria within a 72-h period including the day preceding and the day following IAI diagnosis.

Pneumoniae, other bacteremia (other than from intraabdominal origin), and catheter-related bloodstream infections were considered at diagnosis of IAI if they were diagnosed within 48 h preceding the IAI diagnosis.

### Outcomes

Primary outcome was 30-day mortality. Secondary outcomes were surgical complications, redo laparotomies or percutaneous drainage, postoperative infectious complications, and septic shock at day 30.

### Statistical analysis

Patient characteristics were expressed in numbers (percentages) and median (interquartile interval) for qualitative and quantitative variables, respectively. They were compared using chi-squared and Mann-Whitney tests respectively. The impact on day 30 mortality of *Enterococcus* species and inappropriate IAT on *Enterococcus* species were assessed with univariate Cox models. Then, multivariate Cox models adjusted on SAPS score on day 0, acquisition of peritonitis in ICU, and adequacy of IAT on other germs were used. For every analysis, *p* <  0.05 was considered significant. All statistics were done using SAS software (v 9.3, SAS Institute, Cary, NC, USA).

## Results

Between 1997 and 2016, data of 1017 patients with IAI were analyzed. Only 76 IAI with *Enterococcus* were included (Additional file 1). Incidence of *Enterococcus* differed between centers (Additional file 2). Median [IQR] age was 72 [59–78]. Fifty-seven percent of patients were male. Median SAPS II score at day 0 was 52 [41–64]. Median time between admission in the hospital or in the ICU and diagnosis of IAI was 8 [5–10] and 1 [1–4] days, respectively. Eight (10.5%) patients had a community-acquired IAI. Sixty-eight (89.5%) patients had a nosocomial IAI, among them 2 were healthcare-associated IAI and 66 were hospital-acquired IAI. Two patients had a percutaneous drainage as an initial treatment, and 7 had a percutaneous drainage secondarily after an initial surgical treatment. IAI characteristics are described in Table 1.

**Table 1:** Population characteristics. Results expressed in numbers (percentages) except *median and interquartile interval [1st–3rd]. *IAI* intraabdominal infection

Variables	All IAI with *Enterococcus* spp. (*n* = 76)
Age (years)*	71.7 [59.0–78.1]
Gender (M/F)	43/33
Day 0 SAPS score*	48 [37–57]
Immunocompromised status	20 (26.3%)
Hospital admission-IAI time*	8 [2–18]
ICU admission-IAI time*	1 [1–4]
IAI diagnosis-surgery time	0 [0–0]
*E. faecium*	28 (36.8%)
*E. faecalis*	46 (60.5%)
Other *Enterococcus* spp.	9 (11.8%)
ICU acquired	24 (31.6%)
Nosocomial	68 (89.5%)
Postoperative	53 (69.7%)
Enterococcal bacteremia	4 (5.3%)
Septic shock at time of IAI diagnosis	53 (69.7%)
Source control	
Surgery	74 (97.4%)
Percutaneous drainage	2 (2.6%)
IAI anatomical origin	
Colon	32 (42.7%)
Small intestine	19 (25.3%)
Hepatobiliary	12 (16%)
Gastroduodenal	8 (10.7%)
Pathophysiology of IAI	
Perforation	22 (28.9%)
Intraabdominal abscess	27 (35.5%)
Fistula	26 (34.2%)
Necrosis	19 (25.0%)
Surgical complications	35 (46.1%)
Intraabdominal abscess	20 (26.3%)
Wound infection	19 (25.0%)
Fistula	8 (10.5%)
Suture line disruption	2 (2.6%)
Evisceration	1 (1.3%)
Relaparotomy or percutaneous drainage at day 30	23 (30.3%)
IAI-relaparotomy or IAI-percutaneous drainage time (days)*	10 [6–20]
Tertiary peritonitis	16 (21.3%).
Postoperative infectious complications at day 30	24 (31.6%)
Septic shock at day 30	44 (57.9%)
Mortality at day 30	17 (22.4%)

Germs associated with *Enterococcus* were as follows: Gram-positive cocci (22%), Gram-negative bacilli (74%), anaerobes (20%), and yeasts (24%). IAI were associated with *Enterococcus* bacteremia in 4 (5%) of cases. Eleven (14%) of the 76 IAI were growing with only *Enterococcus* species. Empirical antimicrobial therapies were piperacillin-tazobactam (49%), carbapenems (33%), vancomycin (30%), and third-generation cephalosporins (9%) in combination with aminoglycosides in 70% of the cases. Initial empirical antimicrobial therapy was inappropriate against *Enterococcus* species isolated from peritoneal sample in 18 (23.7%) of cases and against other germs in 12 (15.8%) of cases. Antimicrobial therapy was modified to cover the recovered Enterococci in 13 (72%) patients. Sensitivity to amoxicillin and vancomycin was always available. There were 3 ESBL and no VRE.

**Fig. 1:**
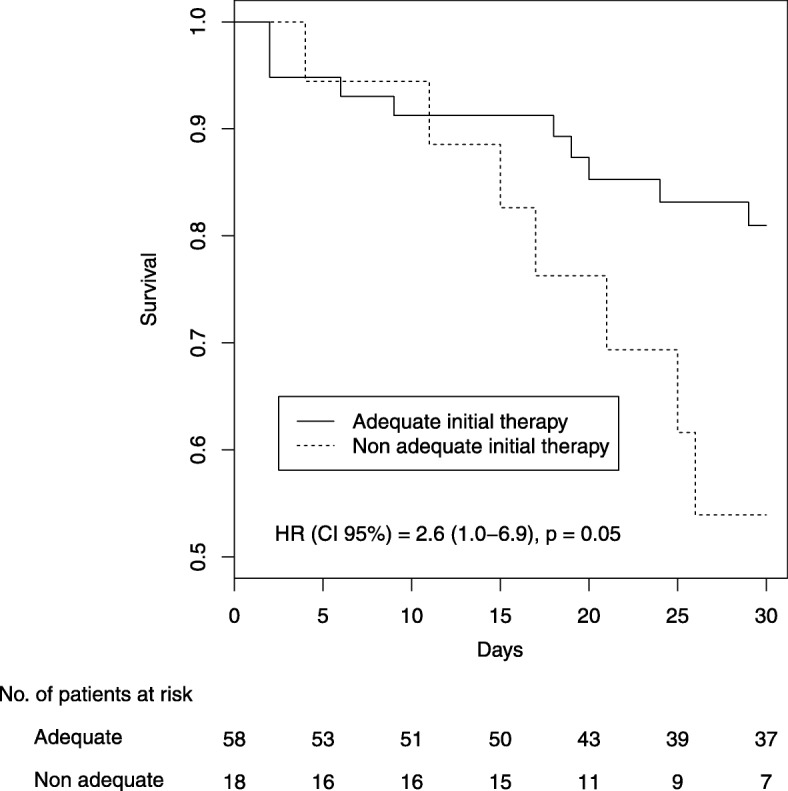
Survival according to adequacy of initial antimicrobial therapy on *Enterococcus* species identified on peritoneal sample (Kaplan-Meier plot)

Table 2 compares patients who received adequate versus inadequate empirical therapy. The two groups were similar for year of inclusion, age, gender, causes, and origins of IAI. Thirty-day mortality was significantly higher in the group who received inadequate empiric antimicrobial therapy against *Enterococcus* species identified on peritoneal sample, but there was no difference in postoperative complications. In this group, *Enterococcus* spp. other than *Enterococcus faecalis* were more frequently identified. Survival curves according to adequacy of IAT against *Enterococcus* species identified on peritoneal sample are shown in Fig. 1.

**Table 2:** Comparison between patients who received an appropriate initial antimicrobial therapy against *Enterococcus* species isolated from peritoneal sample versus patients who did not and between the groups *E. faecalis* alone and *Enterococcus* other than *E. faecalis* alone. Results expressed in numbers (percentages) apart from *median and interquartile interval [1st–3rd]. **Diagnosed within 48 h preceding IAI diagnosis. *IAI* intraabdominal infections

Variables	Initial antimicrobial therapy inactive against *Enterococcus* (*n* = 18)	Initial antimicrobial therapy active against *Enterococcus* (*n* = 58)	*p*	*Enterococcus* other than *E. faecalis* alone (*n* = 34)	*E. faecalis* alone without other *Enterococcus* species (*n* = 42)	*p*
Antimicrobial therapy use < 3 months prior to IAI	9 (50.0)	34 (58.6)	0.519	26 (76.5)	17 (40.5)	0.002
Third-generation cephalosporin use < 3 months prior to IAI	6 (33.3)	7 (12.1)	0.036	9 (26.5)	4 (9.5)	0.051
Hospital admission-IAI time*	4 [2–7]	10 [3–22]	0.016	10 [3–24]	6 [2–13]	0.104
ICU admission-IAI time*	1 [1–4]	1 [1–5]	0.622	2 [1–7]	1 [1–1]	0.024
IAI diagnosis-surgery time*	0 [0–0]	0 [0–0]	0.728	0 [0–0]	0 [0–0]	0.986
Postoperative IAI	7 (38.9)	18 (79.3)	0.001	23 (67.6)	30 (71.4)	0.721
ICU-acquired IAI	8 (44.4)	16 (27.6)	0.179	15 (44.1)	9 (21.4)	0.034
SAPS on day 0*	56.5 [39–63]	45.5 [37–55]	0.029	50.0 [39–60]	45.5 [36–54]	0.051
Day 0 vasopressor use	14 (77.8)	40 (69.0)	0.471	27 (79.4)	27 (64.3)	0.148
Septic shock at diagnosis	15 (83.3)	38 (65.5)	0.151	26 (76.5)	27 (64.3)	0.250
Pneumonia**	1 (5.6)	1 (1.7)	0.375	1 (2.9)	1 (2.4)	0.879
Other bacteremia**	2 (11.1)	4 (6.9)	0.562	2 (5.9)	4 (9.5)	0.558
Catheter-related bloodstream infection**	0 (0)	3 (5.2)	0.151	1 (2.9)	2 (4.8)	0.685
Adequate initial antimicrobial therapy against other germs	14 (77.8)	50 (86.2)	0.392	28 (82.4)	36 (85.7)	0.689
Inadequate initial antimicrobial therapy against identified *Enterococcus*	18 (100)	0 (0)	< 0.001	22 (64.7)	36 (85.7)	0.032
Vancomycin in initial antimicrobial regimen	1 (5.6)	22 (37.9)	0.009			
*E. faecium* isolated in peritoneal sample	11 (61.1)	17 (29.3)	0.015			
*E. faecalis* isolated in peritoneal sample	8 (44.4)	38 (65.5)	0.110			
Other *Enterococcus* isolated in peritoneal sample	2 (11.1)	7 (12.1)	0.913			
Day 30 surgical complications	8 (44.4)	27 (46.6)	0.875	15 (44.1)	20 (47.6)	0.761
Day 30 relaparotomy or percutaneous drainage	4 (22.2)	19 (32.8)	0.395	7 (20.6)	16 (38.1)	0.099
Day 30 infectious complications	7 (38.9)	17 (29.3)	0.445	12 (35.3)	12 (28.6)	0.531
Day 30 mortality	7 (38.9)	10 (17.2)	0.054	12 (35.3)	5 (11.9)	0.015

**Fig. 2:**
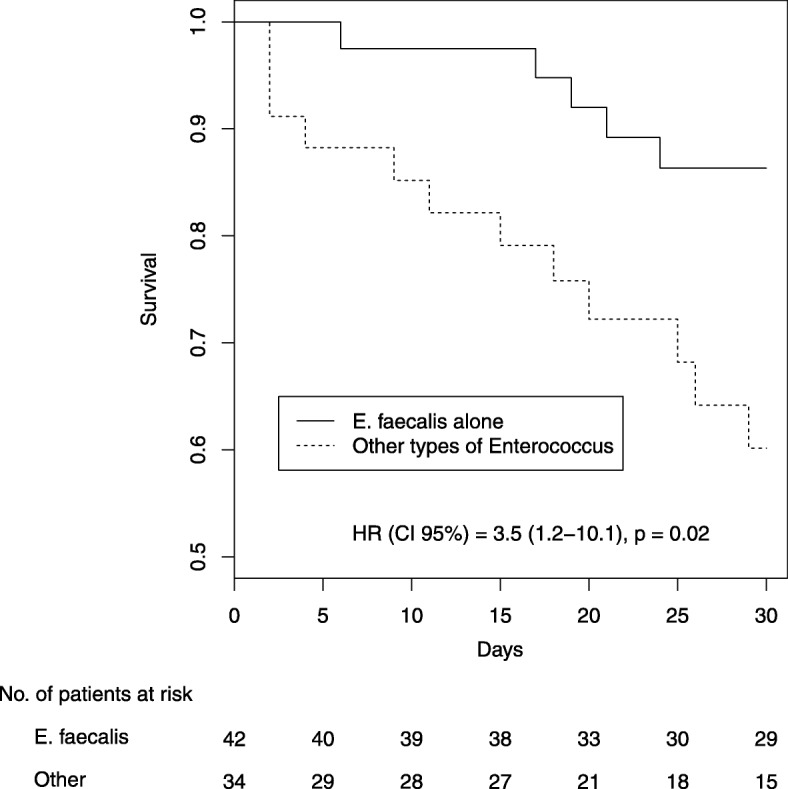
Survival according to *Enterococcus* species (Kaplan-Meier plot)

Comparisons between patients with IAI growing with *Enterococcus* spp. other than *Enterococcus faecalis* and *Enterococcus faecalis* alone are described in Table 2. Day 30 mortality was significantly higher in IAI growing with *Enterococcus* spp. other than *E. faecalis* alone but there was no difference in postoperative complications. Survival curves according to *Enterococcus* species are shown in Fig. 2.

Impact on 30-day survival of the enterococcal species and the adequacy of antibiotic therapy on enterococci is displayed on Table 3.

**Table 3:** Univariate and multivariate models evaluating the impact of *Enterococci* spp. and adequacy of initial antimicrobial therapy on enterococci on day 30 mortality, with adjustment on SAPS score and septic shock at diagnosis, acquisition of IAI in ICU, and adequacy of initial antimicrobial therapy on other germs. *As interaction term between adequate therapy and other than *E. faecalis* alone IAI was significant, we created a variable with three classes, *E. faecalis* IAI was the reference

Parameters	HR	CI 95%		*p* value
Univariate models				
No *E. faecalis* alone	3.545	1.247	10.079	0.018
Inappropriate IAT on *Enterococcus spp.*	2.612	0.991	6.883	0.052
No *E. faecalis* alone + inadequate IAT on *Enterococcus* spp.*	4.427	1.277	15.344	0.019
No *E. faecalis* alone + adequate IAT on *Enterococcus* spp.*	3.106	0.985	9.796	0.053
Multivariate models				
Inappropriate IAT on *Enterococcus* spp.	1.445	0.498	4.195	0.498
SAPS on day 0	1.003	0.964	1.043	0.893
ICU-acquired peritonitis	3.282	1.191	9.041	0.021
Adequate IAT on other germs	1.364	0.299	6.221	0.689
Septic shock at diagnosis	11.828	1.451	96.396	0.021
No *E. faecalis* alone	2.283	0.730	7.141	0.156
SAPS on day 0	1.003	0.964	1.043	0.890
ICU-acquired peritonitis	2.613	0.898	7.605	0.078
Adequate IAT on other germs	1.469	0.312	6.927	0.627
Septic shock at diagnosis	11.101	1.386	88.887	0.023
No *E. faecalis* alone + inadequate IAT on *Enterococcus* spp.	2.290	0.551	9.519	0.254
No *E. faecalis* alone + adequate IAT on *Enterococcus* spp.	2.281	0.690	7.535	0.176
SAPS on day 0	1.003	0.964	1.043	0.890
ICU-acquired peritonitis	2.611	0.859	7.937	0.091
Adequate IAT on other germs	1.470	0.307	7.031	0.629
Septic shock at diagnosis	11.094	1.371	89.776	0.024

Univariate analysis demonstrated that the identification of species other than *E. faecalis* alone was associated with death and the mortality rate was greater if the antibiotic therapy was inadequate against *Enterococcus* spp. identified on peritoneal samples.

However, after adjusting for confounders (i.e., SAPS II and septic shock at IAI diagnosis, ICU-acquired peritonitis, and adequacy of antibiotic therapy for other germs), the impact of the adequacy of antimicrobial therapy was no longer significant (Table 3). The impact of culturing enterococci other than *E. faecalis* remained a poor prognosis (HR = 2.283 [0.730–7.141], *p* = 0.156). A septic shock at IAI diagnosis and an ICU-acquired IAI were associated with death regardless of the adequacy of IAT and *Enterococcus* species.

Survival curves according to adequacy of IAT on germs other than *Enterococcus* species identified on peritoneal sample are shown in Additional file 3.

Neither adequacy of IAT nor *Enterococcus* species were associated with redo laparotomy on day 30 or percutaneous drainage in both univariate and multivariate analyses.

## Discussion

In a large cohort of severe peritonitis with enterococci admitted in the ICU, we found that inadequate IAT against *Enterococcus* spp. was associated with increased 30-day mortality. We also found that *Enterococcus* spp. other than *E. faecalis* alone were more frequent in cases of previous therapy with third-generation cephalosporins in the past 3 months and in ICU-acquired peritonitis. It was associated with inadequate IAT and a poorer prognosis.

Intraabdominal infections growing with *Enterococcus* are associated with a worse prognosis. Theunissen et al. showed that presence of Enterococci is a predictive factor for death in both nosocomial and community-acquired IAI and is independently associated with mortality (OR 3.88 (1.05–14.28) *p* = 0.044) [19]. In our study, the large majority of IAI was nosocomial (90%) and mortality was high (22% in the whole population and 39% in the inappropriate first antimicrobial therapy group). It was comparable with previously published data: 39% in Montravers et al. study (mainly postoperative IAI with multidrug-resistant bacteria), 25% in Sotto et al. (ICU IAI), and 25% in Theunissen et al. (40% of nosocomial IAI) [17, 19, 26]. Recently, Freedberg et al. found in a cohort of 301 medical ICU patients that VRE colonization and *Enterococcus* domination were both associated with death or all-cause infection at 30 days (aHR 1.46, 95% CI 1.06–2.00 and aHR 1.47, 95% CI 1.00–2.19, respectively) after adjusting for severity of illness [27].

We found that an inappropriate IAT against *Enterococcus* spp. identified on peritoneal samples was associated with a higher 30-day mortality. The only prospective randomized trial that compared treatment with antimicrobial therapy active or inactive against *Enterococcus* (penicillin vs cephalosporin) concluded no differences between both groups [28]. This study included only non-severe community-acquired IAI (median APACHE scores 10 and 9); the number of IAI growing with *Enterococcus* was very low (6 over 110 peritonitis) [28]. However, in a population of 200 postoperative IAI among which 42 were growing with *Enterococcus*, Sitges-Serra et al. found that mortality was higher when IAT did not cover these *Enterococcus* (21% vs 4%, *p* <  0.001) [14]. In *Enterococcus* bacteremia, early use of anti-*Enterococcus* antimicrobial therapy within 48 h is a protective factor against death [29].

Several studies have evaluated the impact on mortality of the adequacy of an initial empiric antimicrobial therapy for all peritoneal germs in general (not only for *Enterococcus*). Sotto, Mosdell, Montravers, and Sturkenboom did not find any difference [17, 20, 30, 31]. However, Sotto studied ICU IAI but the numbers were small in the inappropriate antimicrobial therapy group (14 patients) and the three other studies included mainly patients with community-acquired and non-severe IAI. Nevertheless, Montravers’ study on postoperative IAI growing with multiresistant bacteria (2009) and Harbarth’s study on severe sepsis and septic shock showed that an inappropriate IAT was an independent risk factor of death in IAI [26, 32]. Thus, it seems that an appropriate IAT has an impact on mortality especially in severe postoperative IAI with septic shock.

However, we did not find more redo laparotomies or percutaneous drainages or infectious complications at day 30 when IAT was inappropriate. Other studies showed that an inappropriate IAT against all microbials found on the peritoneal sample was associated with a higher morbidity both in community-acquired and nosocomial IAI in terms of length of stay, wound infection, redo laparotomies, and postoperative complications [14, 26, 30, 31, 33, 34].

An inadequate IAT against *Enterococcus* spp. identified on peritoneal samples was associated with postoperative IAI, time between hospital admission and IAI, antimicrobial therapy with third-generation cephalosporins within the 3 months prior to IAI, IAI with *E. faecium* or any *Enterococcus* species other than *E. faecalis*, and an IAT that did not include vancomycin. This implies that adequacy of IAT was associated with the use of vancomycin. WSES recommendations are to cover *Enterococcus* in postoperative IAI but not in community-acquired IAI [35]. For IDSA, “empiric anti-enterococcal therapy is recommended for patients with healthcare-associated IAI, particularly those with postoperative infection, those who have previously received cephalosporins or other antimicrobial agents selecting for *Enterococcus* species, immunocompromised patients, and those with valvular heart disease or prosthetic intravascular materials”. “Anti-enterococcal therapy should be directed against *Enterococcus faecalis*”, and “antibiotics that can potentially be used … include ampicillin, piperacillin-tazobactam or vancomycin” [36].

In multivariate analysis, adequacy of IAT on *Enterococcus* species was no longer independently associated with survival after adjustment on adequacy of IAT on other germs, time of onset of IAI (ICU-acquired IAI), SAPS score, and septic shock at time of diagnosis. However, septic shock at diagnosis and ICU-acquired IAI were risk factors for death regardless of the *Enterococcus* species and adequacy of IAT. It has been shown multiple times in the literature that an IAI with septic shock has a higher mortality rate than an IAI without septic shock [2, 20, 37] and our study confirms this finding.

Finally, we found a difference in 30-day mortality depending on *Enterococcus* species: *E. faecalis* alone vs *Enterococcus* other than *E. faecalis* alone (mostly *E. faecium*). In univariate analysis, we found a higher hazard ratio for death with an *Enterococcus* other than *E. faecalis* alone that had been initially inadequately treated compared to adequately treated; this suggests that the species of *Enterococcus* had a greater impact than adequacy of IAT on survival. Risk factors of having an *Enterococcus* other than *E. faecalis* alone in univariate analysis were SAPS score on day 0, Cardio SOFA score on ICU admission, time between ICU admission and IAI, ICU-acquired IAI, antimicrobial therapy within the 3 months prior to IAI especially with third-generation cephalosporins, antimicrobial therapy prior to relaparotomy in postoperative IAI, and an inadequate IAT. Previous studies on enterococcal bacteremia found that SAPS score, prior antimicrobial therapy exposure (mainly penicillin and third-generation cephalosporins, but also carbapenems, aminoglycosides, and clindamycin), hematologic malignancies and neutropenia, current corticosteroid therapy, organ dysfunction, gastrointestinal disease (vs genitourinary disease), and nosocomial acquisition were risk factors for *E. faecium* isolation (vs *E. faecalis*) [38–42]. Some studies found a higher mortality with *E. faecium* than with *E. faecalis* bloodstream infections [38, 40, 42]. Our findings are consistent with those studies. *Enterococcus* spp., especially other than *E. faecalis*, are associated with a poorer prognosis in IAI and their presence is often associated with a previous antimicrobial therapy within the 3 months prior to IAI and especially with third-generation cephalosporins.

### Our study has several limits

First, the number of patients especially in the inappropriate IAT group was low. We can explain the small number of patients by the very selected population we studied. But to our knowledge, this is the second largest cohort of IAI growing with *Enterococcus* after Kaffarnik et al.’s cohort [21]. However, because the population is highly selected, the results of our study should only be taken as an exploratory approach and further studies are needed to confirm those results.

Second, this is a retrospective study although data was extracted from a multicentric national database in which data are collected prospectively. Presence of missing data led us to read medical records, but since almost one third of medical records were not available, we had to exclude nearly a hundred of patients. Information bias cannot be excluded especially about previous antimicrobial therapy before hospital admission.

Third, we had no prospectively collected data about the quality of source control. All patients had either surgery or percutaneous drainage. Yet it is known that a good quality of source control is associated with a reduction in mortality in IAI [43]. Therefore, it is a limitation to conclude on mortality without information about source control quality. However, evaluation would have been subjective since there is no validated questionnaire to evaluate source control quality apart from the checklist recommended by Solomkin et al. to be done at the end of surgery or percutaneous drainage [44].

Finally, data collection was extended over 20 years. Both surgery and critical care evolved during this period. Critical care management improved widely for the last 10 years which led to a better prognosis in sepsis. However, there were no difference in years of inclusion between groups.

## Conclusion

We found that inadequacy of IAT on *Enterococcus* species identified on peritoneal sample was associated with a worse 30-day survival especially when it was not an *E. faecalis* alone. This inadequacy was partly due to the absence of vancomycin in the empiric antibiotic regimen. These results should encourage the physician to use an antimicrobial regimen active against non-*faecalis Enterococcus* (like vancomycin) in severe, postoperative, ICU-acquired IAI, especially when an antimicrobial therapy with third-generation cephalosporins has been used within 3 months preceding IAI. Our study also suggests that IAI with non *E. faecalis* Enterococci have a poorer prognosis than IAI with *E. faecalis* alone. It would be interesting to conduct a prospective and larger study to confirm these results.

## Additional files

Additional file 1: Flowchart. IAI = intraabdominal infection. (DOCX 46 kb)

Additional file 2: Incidence of *Enterococcus* in each center. IAI = intraabdominal infection. (DOCX 13 kb)

Additional file 3: Survival rate according to adequacy of initial antimicrobial therapy on germs other than *Enterococcus* species identified on peritoneal sample (Kaplan-Meier plot, *n* = 64). (PDF 25 kb)

## Data Availability

The datasets generated and/or analyzed during the current study are available in the OutcomeRea database.

## Abbreviations

IAI: Intraabdominal infection
IAT: Initial antimicrobial therapy
GCS: Glasgow Coma Scale
SAPS II: Simplified Acute Physiology Score II
SOFA: Sequential Organ Failure Assessment
