# Supplementary figures and images for: Impact of species and antibiotic therapy of enterococcal peritonitis on 30-day mortality in critical care—an analysis of the OUTCOMEREA database
Source: Crit Care. 2019 Sep 6;23:307. doi: 10.1186/s13054-019-2581-8 (PMC6731585; doi:10.1186/s13054-019-2581-8)

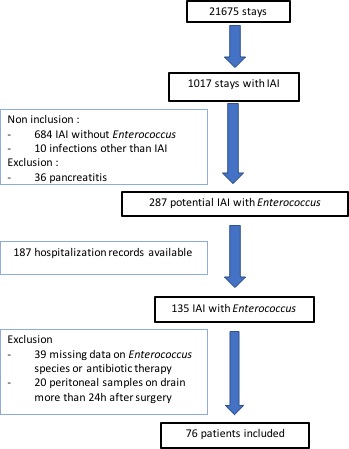

Supplement: Supplementary file 1 — Flowchart. IAI = intraabdominal infection. (DOCX 46 kb) [file 13054_2019_2581_MOESM1_ESM.docx]

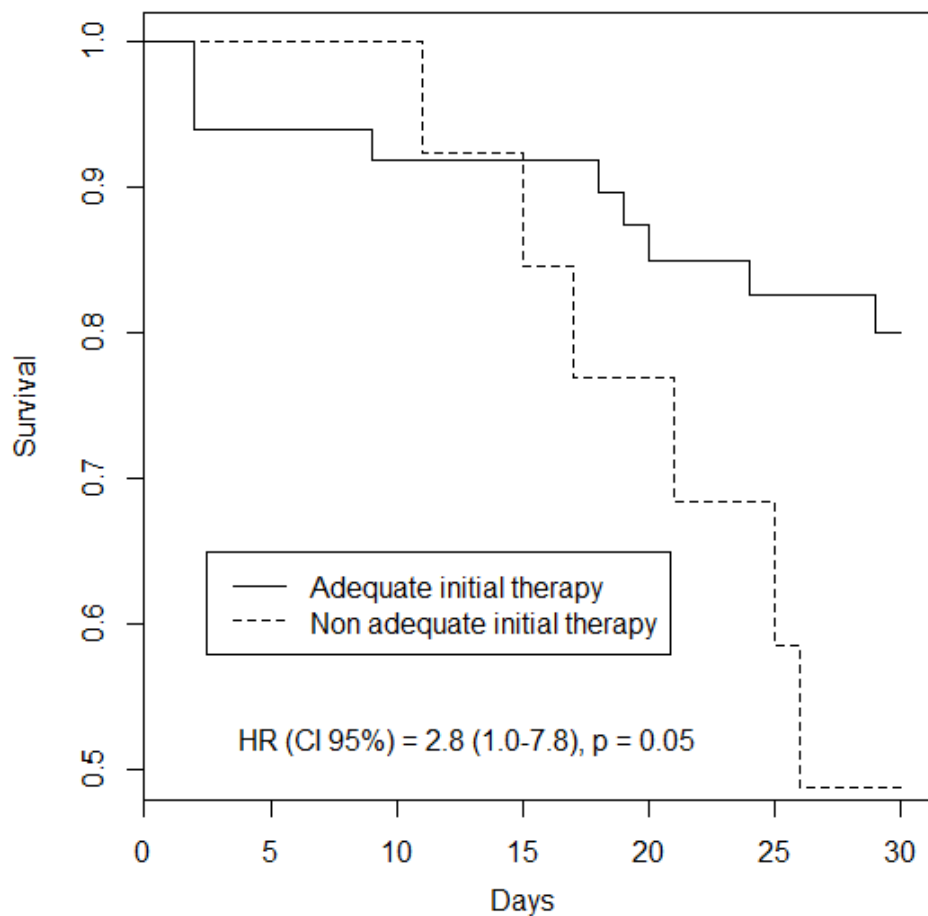

No. of patients at risk

|              |    |    |    |    |    |    |    |
|--------------|----|----|----|----|----|----|----|
| Adequate     | 50 | 45 | 44 | 43 | 37 | 34 | 32 |
| Non adequate | 14 | 13 | 13 | 12 | 9  | 7  | 5  |

Supplement: Supplementary file 3 — Survival rate according to adequacy of initial antimicrobial therapy on germs other than Enterococcus species identified on peritoneal sample (Kaplan-Meier plot, n = 64). (PDF 25 kb) [file 13054_2019_2581_MOESM3_ESM.pdf]
