# Supplementary material for: Impact of species and antibiotic therapy of enterococcal peritonitis on 30-day mortality in critical care—an analysis of the OUTCOMEREA database
Source: Crit Care. 2019 Sep 6;23:307. doi: 10.1186/s13054-019-2581-8 (PMC6731585; doi:10.1186/s13054-019-2581-8)
Supplement: Supplementary file 2 — Incidence of Enterococcus in each center. IAI = intraabdominal infection. (DOCX 13 kb) [file 13054_2019_2581_MOESM2_ESM.docx]

Additional file 2:

| Center | Number of patients with IAI  (n=1017) | Number of patients with *Enterococcus* (n=287) | % *Enterococcus* |
| --- | --- | --- | --- |
| Avicenne | 24 | 4 | 17% |
| Bichat | 18 | 8 | 44% |
| Orléans | 1 | 0 | 0% |
| Cayenne | 3 | 0 | 0% |
| Clamart | 140 | 51 | 36% |
| Clermont-Ferrand | 14 | 2 | 14% |
| Colombes | 25 | 9 | 36% |
| Croix St Simon | 5 | 2 | 40% |
| Dourdan-Etampes | 23 | 5 | 22% |
| Gonesse | 39 | 9 | 23% |
| Grenoble | 202 | 69 | 34% |
| HM Nord | 5 | 2 | 40% |
| Hôtel Dieu | 1 | 0 | 0% |
| Hyères | 2 | 0 | 0% |
| Lyon surgical ICU | 56 | 11 | 20% |
| Lyon medical ICU | 8 | 1 | 13% |
| Melun | 2 | 0 | 0% |
| Nantes | 5 | 1 | 20% |
| Henri Mondor CCU | 1 | 0 | 0% |
| St Denis | 31 | 1 | 3% |
| St Etienne | 9 | 2 | 22% |
| St Joseph surgical ICU | 29 | 1 | 3% |
| St Joseph medical ICU | 299 | 88 | 29% |
| St Louis | 47 | 10 | 21% |
| Tenon | 7 | 0 | 0% |
| Timone | 3 | 0 | 0% |
| Versailles | 18 | 11 | 61% |
